# Inhibition of FASN suppresses the malignant biological behavior of non-small cell lung cancer cells via deregulating glucose metabolism and AKT/ERK pathway

**DOI:** 10.1186/s12944-019-1058-8

**Published:** 2019-05-24

**Authors:** Ligong Chang, Surong Fang, Yubao Chen, Zhenhua Yang, Yuan Yuan, Jing Zhang, Liang Ye, Wei Gu

**Affiliations:** Department of Respiratory Medicine, Nanjing First Hospital, Nanjing Medical University, No. 68 Changle Road, Qinhuai District, Nanjing, 210001 People’s Republic of China

**Keywords:** NSCLC, Fatty acid synthase, AKT/ERK pathway, Glucose metabolism, Xenograft

## Abstract

**Background:**

Fatty acid synthase (FASN) is overexpressed in most human carcinomas, including non-small cell lung cancer (NSCLC), and contributes to poor prognosis. An increasing number of studies have highlighted the potential function of FASN as both a biomarker and therapeutic target for cancers. However, the underlying molecular mechanisms of FASN in glucose metabolism and the malignant biological behavior of NSCLC remain the subjects of intensive investigation.

**Methods:**

FASN expression was depleted by FASN-siRNA in A549 and NCI-H1299 cell lines to detect the function of glucose metabolism and the malignant biological behavior of NSCLC cells. Western-blot and qPCR were applied to determine the expressions of FASN, t-AKT, p-AKT, t-ERK, p-ERK, PKM2, HK2 and AZGP1. ATP and lactate were detected to determine the activation of glucose metabolism. CCK8 and transwell assays were used to detect the proliferation, invasion, and migration capacity of the two types of NSCLC cells. The xenograft mouse model was used to evaluate tumor weights after suppression of FASN.

**Results:**

LV-FASN-siRNA and its control lentiviral vector were successfully transfected into the two types of NSCLC cells (A549 and NCI-H1299). LV-FASN siRNA significantly suppressed FASN expression in both NSCLC cell types, and expressions of p-AKT, p-ERK, PKM2, and AZGP1 were also significantly decreased. Notably, the levels of ATP and lactate were significantly decreased after transfection with LV-FASN siRNA. The proliferation of both NSCLC cell types was decreased after suppression of FASN. The invasion and migration capacity of A549, but not NCI-H1299, were inhibited following down-regulation of FASN. In vivo, inhibition of FASN caused a marked animal tumor weight loss.

**Conclusions:**

FASN was involved in glucose metabolism via down-regulation of the AKT/ERK pathway and eventually altered the malignant phenotype in lung cancer cells.

## Background

Lung cancer is currently one of the most frequently occurring cancers and is the leading cause of cancer-related death in the world. Non-small cell lung cancer (NSCLC) is a heterogeneous class of tumors that account for approximately 85% of all lung cancer cases globally [1]. Despite rapid developments in therapeutic strategies for NSCLC, the five-year survival rate and final prognosis for NSCLC patients remain very poor. Therefore, understanding the molecular mechanisms behind NSCLC would be of great benefit for its early diagnosis and treatment.

Metabolic reprogramming has received increasing amounts of attention as a hallmark of human cancers [2]. The enhancement of glucose metabolism in cancer cells provides sufficient ATP and numerous carbon intermediates for the biosynthesis of lipids, amino acids, and nucleotides in most human cancers [3]. Additionally, overactive lipid metabolism provides the material basis for the proliferation and migration of cancer cells [4]. Numerous cancer cells undergo exacerbated endogenous fatty acid biosynthesis. A key biosynthetic enzyme of de novo fatty acid synthesis, FASN is over-expressed in most tumors and its activity is required for the malignant biological behavior of tumor cells. Moreover, over-expressed FASN also contributes to unfavorable prognoses and treatment resistance in various tumor types, including lung, bladder, prostate, ovarian, osteosarcoma, breast, colorectal, pancreatic and lymphoma [5–14]. FASN was negatively expressed in 57% (61/106) of NSCLC patients and FASN expression in stage I NSCLC has been reported to be associated with poor outcomes [15, 16]. However, the relationship between FASN and glucose metabolism in NSCLC is largely unknown.

FASN expression is regulated by SREBP-1c, NF-Y, EGCG, AZGP1, NAC1, P300 acetyltransferase, and USP2a isopeptidase. These regulators are modulated by PI3K/AKT/mTOR, ERK/MAPK, Wnt/β-catenin, and protein kinase C signaling cascades [17–20]. The expression of FASN is down-regulated after inhibited Akt/mTOR pathway.[21] Additionally, the proliferation of cancer cells is down-regulated after treatment with different FASN inhibitors [22–24] and suppression of FASN expression inhibits the proliferation and migration of colorectal cancer cells via VEGF and VEGFR-2.[25] It is noteworthy that the activity of the PI3K/AKT/mTOR pathway plays an important role in cellular glucose metabolism.[26, 27] Consistently, activation of the ERK/MAPK pathway has been reported to up-regulate the expression of some essential enzymes involved in glucose metabolism such as PKM2 and HK2.[28, 29] These findings demonstrate that there may be molecular interactions between FASN and its upstream signaling pathway and/or glucose metabolism. Accordingly, in the current study, it is hypothesized that inhibition of FASN will suppress the malignant biological behavior of NSCLC cells via deregulation of glucose metabolism and the AKT/ERK pathway.

## Materials and methods

### Cell lines and cell culture

Two types of classic human NSCLC cell lines (A549 and NCI-H1299) were used in this study and were obtained from the Institute of the Chinese Academy of Sciences (Shanghai, China). The A549 and NCI-H1299 cells were cultured in RPMI-1640 medium (Invitrogen, Thermo Fisher Scientific, USA) plus penicillin G (100 U/ml, Beyotime, China), streptomycin (100 μg/ml, Corning, China) and 10% fetal bovine serum (Hyclone, Life Sciences, Shanghai, China). The cells were incubated in an incubator (Thermo, Waltham, MA, USA) at 37 °C in a humidified atmosphere of 5% CO_2_ and 95% air.

### FASN-siRNA transfection

Lentiviral vectors constructed for FASN small hairpin RNA were purchased from Shanghai Genechem Co., Ltd. (Shanghai, China). This double-stranded siRNA was synthesized according to the manufacturer’s instructions and targeted for AACCCTGAGATCCCAGCGCTG. FASN-siRNA negative control is a nonspecific control pool (Shanghai Genechem Co., Ltd). Briefly, FASN RNAi lentiviral vectors and control lentiviral vectors were mixed with infection solution and added to the cell suspension. The cells were continuously incubated for 48 h, following which the cell culture medium was charged by adding polybrene (5 μg/ml). Green fluorescence (GFP) was detected by fluorescence microscopy (Nikon, TE2000, Tokyo, Japan) to determine the transfection efficiency. Cells were classified into three groups: 1) a blank group (wild-type A549 and NCI-H1299 cells); 2) a control group (A549 and NCI-H1299 cells transfected by FASN-siRNA negative control); and 3) an experimental group (A549 and NCI-H1299 cells transferred by FASN-siRNA).

### Total mRNA isolation and RT-qPCR

Total cellular RNA was isolated using TRIzol reagent (Invitrogen, USA), dissolved in RNA-free H_2_O and stored at − 80 °C. RNA (1 μg) was used to obtain high-quality cDNA using the Hiscript™ first strand cDNA synthesis kit (Vazyme, Nanjing, China) according to the manufacturer’s protocols. The PCR system was used to amplify all transcripts using the AceQTM qPCR SYBR® Green kit. A 20 uL reaction volume was prepared from cDNA (2 μL), SYBR® Green Master Mix (10 μL), upstream and downstream primer (0.8 μL) and H_2_O (7.2 μL). The primer sequences were as follows: FASN (sense 5′-CGACAGCACCAGCTTCGCCA-3′, antisense 5′-CACGCTGGCCTGCAGCTTCT-3′), Akt (sense 5′-AAGCACCGCGTGACCATGAA-3′, antisense 5′-TCTTAATGTGCCCGTCCTTG-3′), AZGP1 (sense 5′-CAACCCTTGCTTCCTAGCTG-3′, antisense 5′-ACCACCAATGCCAAAGTAGCACC-3′), ERK (sense 5′-GCTCACCCTTACCTGGAACA-3′, antisense 5′-GGACCAGATCCAAAAGGACA-3′), and GAPDH (sense 5′-GAAGGTCGGAGTCAACGGATT-3′, antisense 5-CGCTCCTGGAAGATGGTGAT-3′). qPCR was designed with the following conditions: predenaturation at 95 °C for 5 mins followed by 35 cycles of denaturation at 95 °C for 10 s, annealing at 60 °C for 30 s and elongation at 72 °C for 30 s. Target gene mRNA expression was verified and analyzed with the StepOnePlus Real-Time PCR system using GAPDH as an internal standard. Relative transcripts were calculated using the 2^−ΔΔ^CTmethod. All experiments were repeated in triplicate.

### Protein isolation and Western-blot

The three different cell groups were harvested and digested using RIPA lysis buffer (500 μl, Beyotime, Shanghai, China). The samples were then centrifuged at 14,000 g for 15 min at 4 °C. Protein concentrations were determined using BCA kits (Beyotime, Shanghai, China). 2× sample loading buffer was added to the protein samples and the mixture heated at 95 °C for 5 min. Equal amounts of proteins were resolved by electrophoresis in 10% SDS-polyacrylamide gel and then transferred onto NC membranes (Immobilon, Millipore, Bedford, MA). The membranes were blocked with 5% skimmed milk at RT for 1 h, followed by incubation overnight at 4 °C with primary antibodies (anti-FASN, anti-tEKR, anti-pERK^Thr202/Tyr204^, anti-tAKT, anti-pAKT^ser473^, anti-AZGP1, anti-PKM2, anti-HK2, and anti-GAPDH) at a 1:2000 dilution. The membranes were washed with TBST three times and incubated with goat anti-rabbit IgG antibody (Santa Cruz, CA, 1:5000) conjugated with HRP at RT for 1 h. Immunoreactivity was determined using the enhanced ECL Plus™ chemiluminescence kit (Beyotime, Shanghai, China) and an imaging and analysis system.

### Cell viability assay

The cell viabilities of A549 and NCI-H1299 cells after FASN-siRNA transfection were assessed using the Cell Counting Kit-8 assay (Kaiji, Nanjing, China). The different cell groups were suspended at 1000 cells/well in RPMI1640 complete medium (100 μl), then transfected with FASN-siRNA and control FASN-siRNA. At 0, 1, 2, 3, 4, and 5 days after transfection, CCK-8 (10 μl) was added into each well and the plate was incubated for 1 h at 37 °C. Absorbance at 450 nm was measured using an ELISA plate reader (Thermo, Waltham, MA, USA). The OD_450_ value was inversely proportional to the degree of cell proliferation. Each group comprised three duplicated wells, and the assays were performed three times independently.

### Transwell migration and invasion assay

Cell migration or invasion assays were performed using a 24-well Boyden chamber with Matrigel. Briefly, 1 × 10^4^ target cells transfected with FASN-siRNA were plated onto the upper chamber. The lower chamber was filled with RPMI-1640 medium containing 10% fetal bovine serum. The transwell invasion system was incubated at 37 °C for 24 h. The cells on the surface of the basement membrane were wiped off and the lower surfaces of the cells were fixed by cool acetone then stained with crystal violet. The numbers of invaded cells were counted under a microscope. The experiment was performed in triplicate.

### ATP and lactic acid assay

To detect the impact of FANS-RNAi on glucose metabolism, the levels of ATP and lactic acid were detected using the appropriate kits. ATP assay kits and lactic acid assay kits were purchased from Nanjing Jian-cheng Bioengineering Institute (Nanjing, China). Briefly, after A549 and NCI-H1299 transfection by FASN-siRNA, six groups of cells were harvested and homogenized in the cell lysates. The standard sample was diluted by a gradient to make a standard curve using a fluorescence microplate reader (BioTek model FLx800, CA). The ATP and lactic acid computation formulas are as follows:

$$ ATP=\frac{\mathrm{ODtest}-\mathrm{ODcontrol}}{\mathrm{ODstandard}-\mathrm{ODero}}\times \mathrm{standard}\ \mathrm{sample}\times \frac{\mathrm{sample}\ \mathrm{dilution}\ \mathrm{multiple}}{\mathrm{protein}\ \mathrm{concentration}}\left(\mathrm{grot}/\mathrm{L}\right) $$

$$ \mathrm{Lactic}\ \mathrm{acid}=\frac{\mathrm{ODtest}-\mathrm{ODzero}}{\mathrm{ODstandard}-\mathrm{ODzero}}\times \mathrm{standard}\ \mathrm{sample}\times \mathrm{sample}\ \mathrm{dilution}\ \mathrm{multiplier}. $$

The experiments were performed in triplicate.

### Xenograft mouse model

All experiments were approved by the Experimental Animal Ethics Committee of Southeast University (No: 20170104002). Six-week-old nu/nu mice were purchased from the Model Animal Research Center of Nanjing University, China. The mice were raised under SPF circumstances, and all animal experiments complied with WHO guidelines for the humane use and care of animals. A preliminary experiment was performed to detect cell tumorigenicity, wherein wild A549 and NCI-H1299 cells were harvested and resuspended in pre-cooled PBS, following which 10^6^ target cells were injected into the left armpits of the nu/nu mice. The A549 cells were selected and used for subsequent experiments due to the poor tumorigenicity of the NCI-H1299 cells. The three groups of A549 cells were subcutaneously injected into the nu/nu mice. The mice were sacrificed after 2 weeks and their tumors were weighed.

### Statistical analysis

In vitro experiments were confirmed by at least three independent experiments and analyzed by Student’s *t*-tests or one-way ANOVAs in SPSS software (version 13.0; SPSS, Inc., USA), multiple comparison between the groups was performed using S-N-K method. All data are presented as mean ± SD. Xenograft mouse experiment results were analyzed using tumor weights. Data are the median tumor volumes of five animals. Statistical significance was determined at *P* < 0.05 and *P* < 0.01.

## Results

### Inhibition of FASN expression by lentiviral vectors-FASN-RNAi of two NSCLC cell lines

**Fig. 1:**
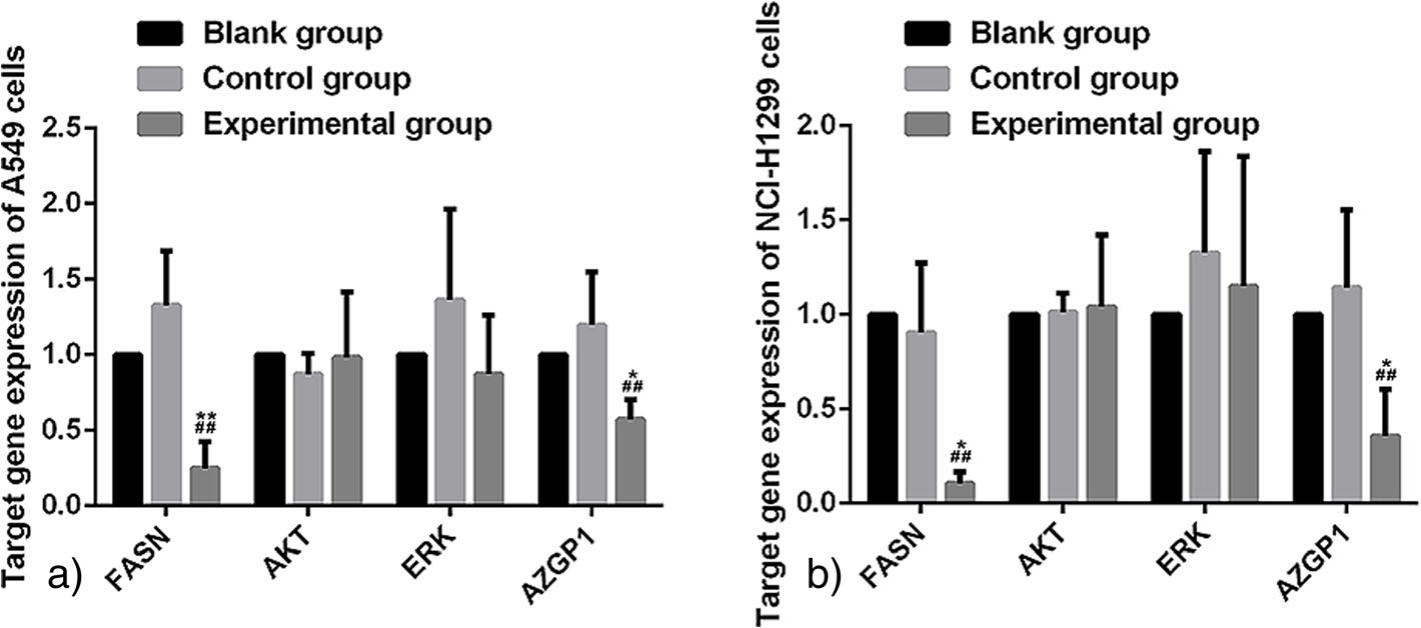
Target genes’ relative expressions detected by RT-qPCR after LV–siFASN transfection. The relative expressions of target genes in each group are shown relative to those of the blank group. FASN mRNA expressions were significantly decreased in the experimental group of A549 cells (*P* < 0.05; **a**) and NCI-H1299 cells (*P* < 0.05; **b**), suggesting that LV–FASN RNAi successfully suppressed the expression of FASN. Additionally, AZGP1 expression was lower under FASN deficiency in A549 cells (*P* < 0.05; a) and NCI-H1299 cells (*P* < 0.05; b). The differences between the Akt-mRNA and ERK-mRNA expressions of each group of A549 cells (**a**) and NCI-H1299 cells (**b**) were not statistically significant. * compared to the control group (*P* < 0.05). # compared to the blank group (*P* < 0.05)

To specifically interfere with the FASN gene, A549 and NCI-H1299 cells were transfected separately with LV-FASN-siRNA to target FASN mRNA. The two cell types were also transfected with LV-control-siRNA to target nonspecific mRNA. Fluorescence (GFP) was detected by an inverted fluorescence microscope (100×) after 48 h transfection to determine transfection efficiency. The relative expressions of target genes were detected by RT-qPCR. The relative expressions of FASN mRNA for the experimental group’s A549 and NCI-H1299 cells were found to be 0.25 ± 0.17 and 0.11 ± 0.06, respectively. These values were significantly lower than those of blank group cells (both *P* = 0.00) and control group cells (1.32 ± 0.36, *P* = 0.00 and 0.91 ± 0.37, *P* = 0.02, respectively). Additionally, the relative expressions of AZGP1 mRNA for the experimental group were 0.57 ± 0.13 and 0.35 ± 0.25 for A549 and NCI-H1299 cells, respectively. These values were also significantly lower than those of the blank group’s A549 and NCI-H1299 cells (*P* = 0.00 and *P* = 0.01, respectively), and the control group’s cells (1.19 ± 0.35, *P* = 0.045 and 1.14 ± 0.41, *P* = 0.047, respectively; Fig. 1ab). However, no significant difference in Akt and ERK mRNA expressions were observed between the A549 and NCI-H1299 cells of the three groups.

### Inhibition of FASN expression suppresses the activity of Akt/ERK signaling pathway

**Fig. 2:**
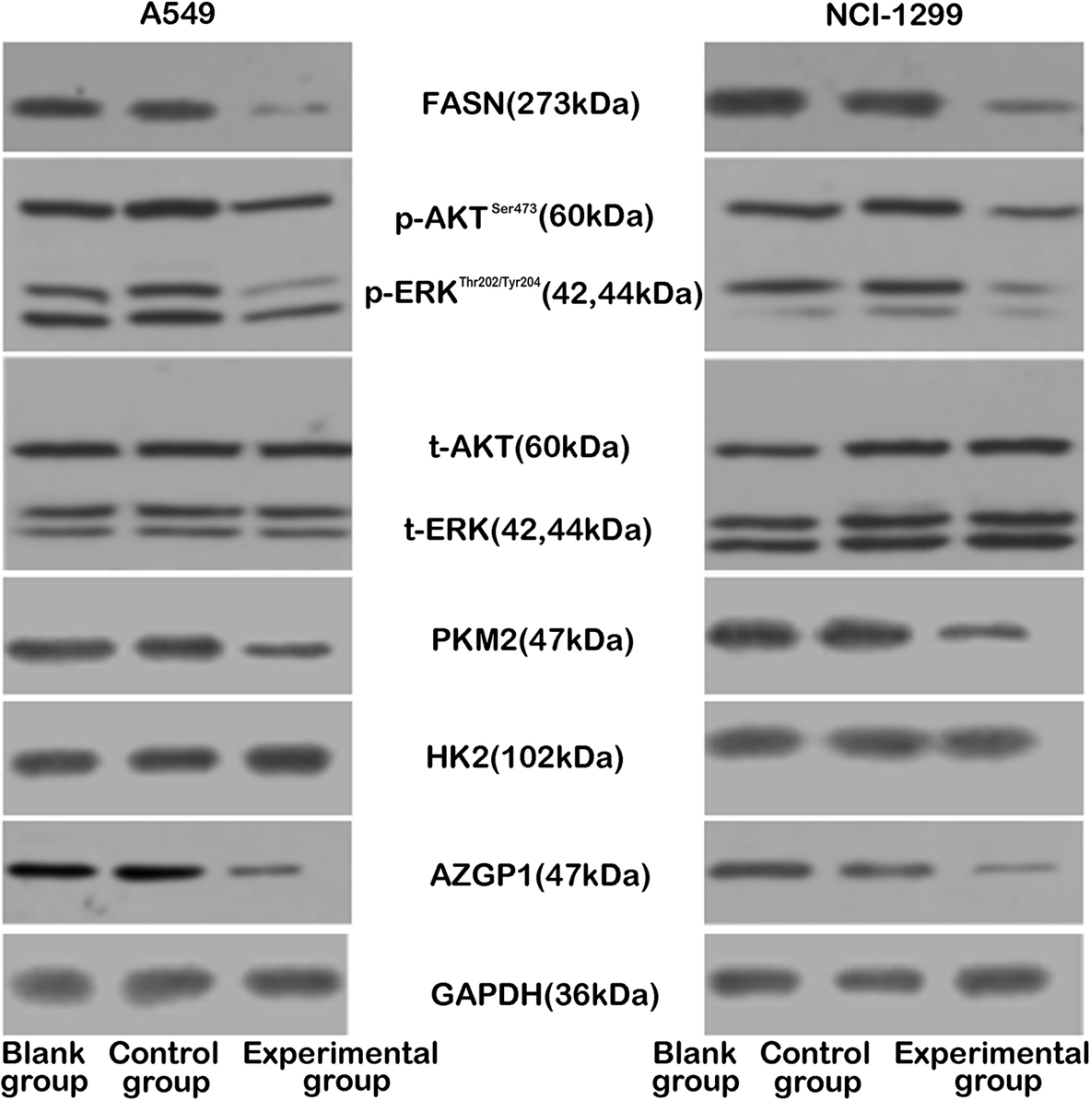
Inhibition of FASN expression suppresses the activity of the Akt/ERK signaling pathway. Target protein expression of A549 cells (left panel) and NCI-H1299 cells (right panel) were detected by western-blot. Compared to the blank and control groups, the expressions of p-Akt and p-ERK in the experimental group were significantly inhibited. However, there were no statistically significant differences in the t-Akt and t-ERK expressions of each group. Importantly, the expression of PKM2 but not HK2 (both are key enzymes in glucose metabolism) was dramatically lower in the experimental group. * compared to the control group (*P* < 0.05). # compared to the blank group (*P* < 0.05)

Target protein expressions of A549 and NCI-H1299 cells in the different treatment groups were measured by western-blot after inhibition of FASN. The expression of FASN was significantly suppressed in the two experimental groups compared to the other groups. Inhibition of FASN expression also significantly inhibited phosph-Akt and phosph-ERK in the A549 and NCI-H1299 cells of the experimental groups compared to those of the two blank and two control groups. Inhibition of FASN also substantially decreased the expression of PKM2, a key enzyme in glucose metabolism, while the expression of HK2, another key enzyme in glucose metabolism, was not significantly decreased. In addition, suppression of FASN dramatically decreased the expression of AZGP1, which is a multifaceted protein associated with lipid mobilization and is regulated by metabolic pathways. However, no significant differences in total-Akt and total-ERK expressions were found any group of A549 and NCI-H1299 cells (Fig. 2).

### Suppression of FASN decreases the activity of glucose metabolism in two NSCLC cell lines

**Fig. 3:**
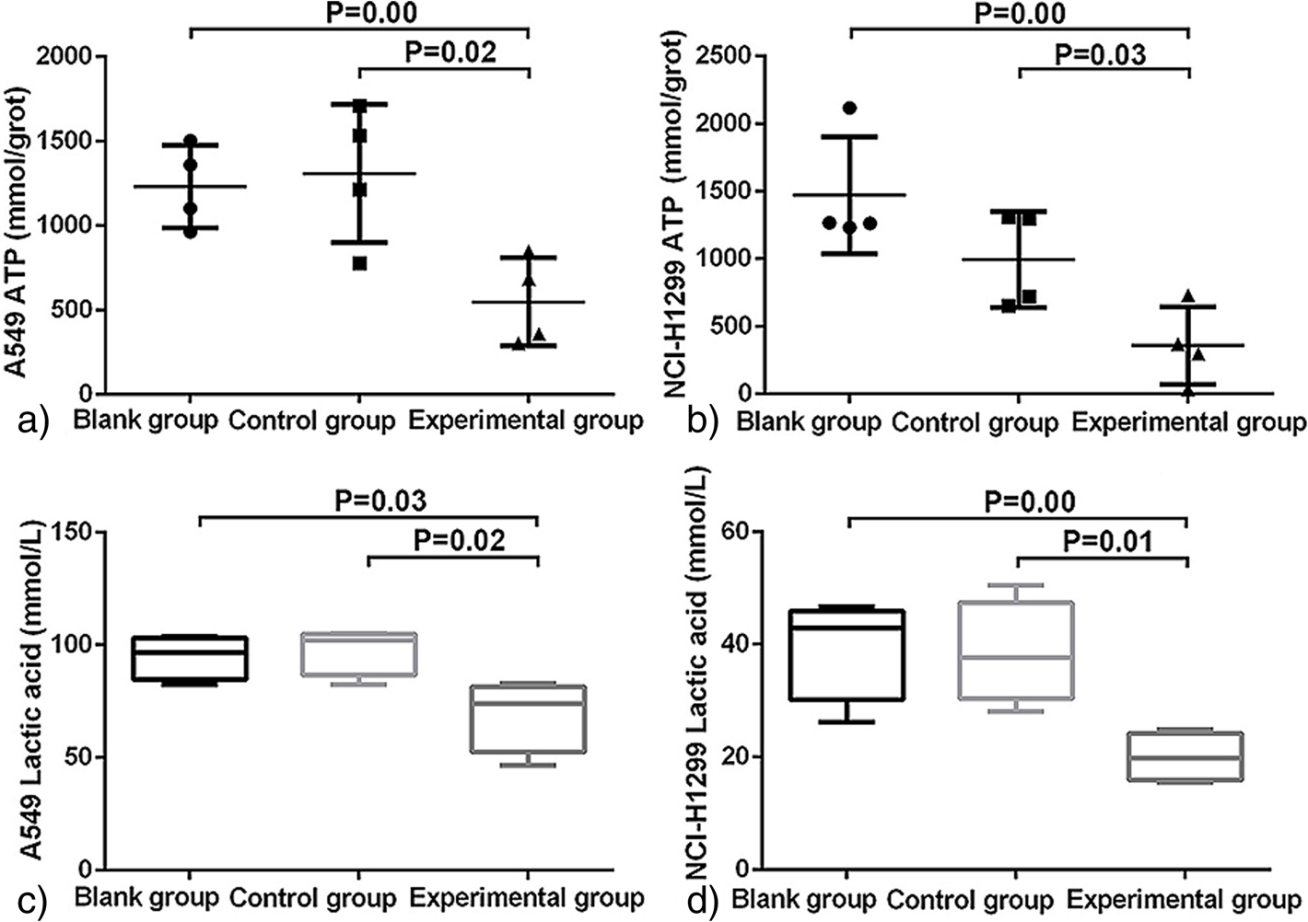
Inhibition of FASN suppresses the activity of glucose metabolism. The levels of ATP (**a**-**b**) and lactic acid (**c**-**d**) were detected by the corresponding kits. ATP levels in the experimental group were significantly lower than those of the blank and control groups (*P* < 0.05). The concentration of lactic acid in the experimental group was also lower in the two types of NSCLC cell lines

To better understand the effects of FASN on glucose metabolism, the levels of ATP and lactic acid were detected after FASN deficiency. The ATP concentration for A549 experimental group cells was 548.75 ± 225.46 mmol/grot, which was significantly lower than for wild-type A549 cells (1232.88 ± 212.18 mmol/grot, *P* = 0.00) and the A549 control group (1308.63 ± 354.99 mmol/grot, *P* = 0.02). A lower ATP concentration (358.56 ± 249.07 mmol/grot) was also found in NCI-H1299 cells of the experimental group compared with those of the blank and control groups (*P* = 0.00 and *P* = 0.03, respectively; Fig. 3a, b). The lactic acid concentration in A549 cells of the experimental group was 69.38 ± 16.07 mmol/L, which was significantly lower than for those of the blank group (94.86 ± 9.65 mmol/L, *P* = 0.03) and control group (97.87 ± 10.54 mmol/L, *P* = 0.02). Similar lactic acid results were also found for NCI-H1299 cells (Fig. 3c, d). These findings suggested that FASN participates in the regulation of glucose metabolism in the two NSCLC cell lines. In combination with the findings of the previous part of the study, these results show that inhibition of FASN expression down-regulates the activity of glucose metabolism via the Akt/ERK/PKM2 pathway.

### Inhibition of FASN suppresses the malignant biological behavior of NSCLC cells

**Fig. 4:**
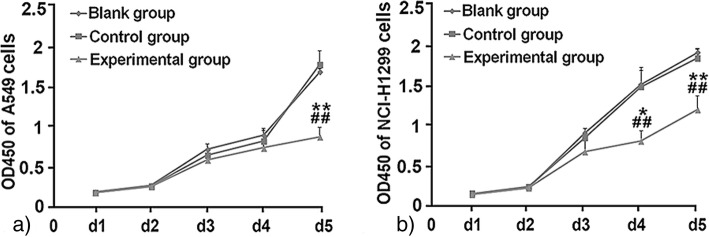
Inhibition of FASN suppresses the proliferation of NSCLC cells. Cell viability assays were conducted 1–5 days after RNAi transfection. Compared with the blank and control groups, the proliferation of experimental group A549 cells was significantly lower at the fifth day post-transfection (**a**); and experimental group NCI-H1299 cells were significantly lower 4 days post-transfection (**b**). * *P* < 0.05, ** *P* < 0.01: compared to the control group. # *P* < 0.05, ## *P* < 0.01: compared to the blank group

**Fig. 5:**
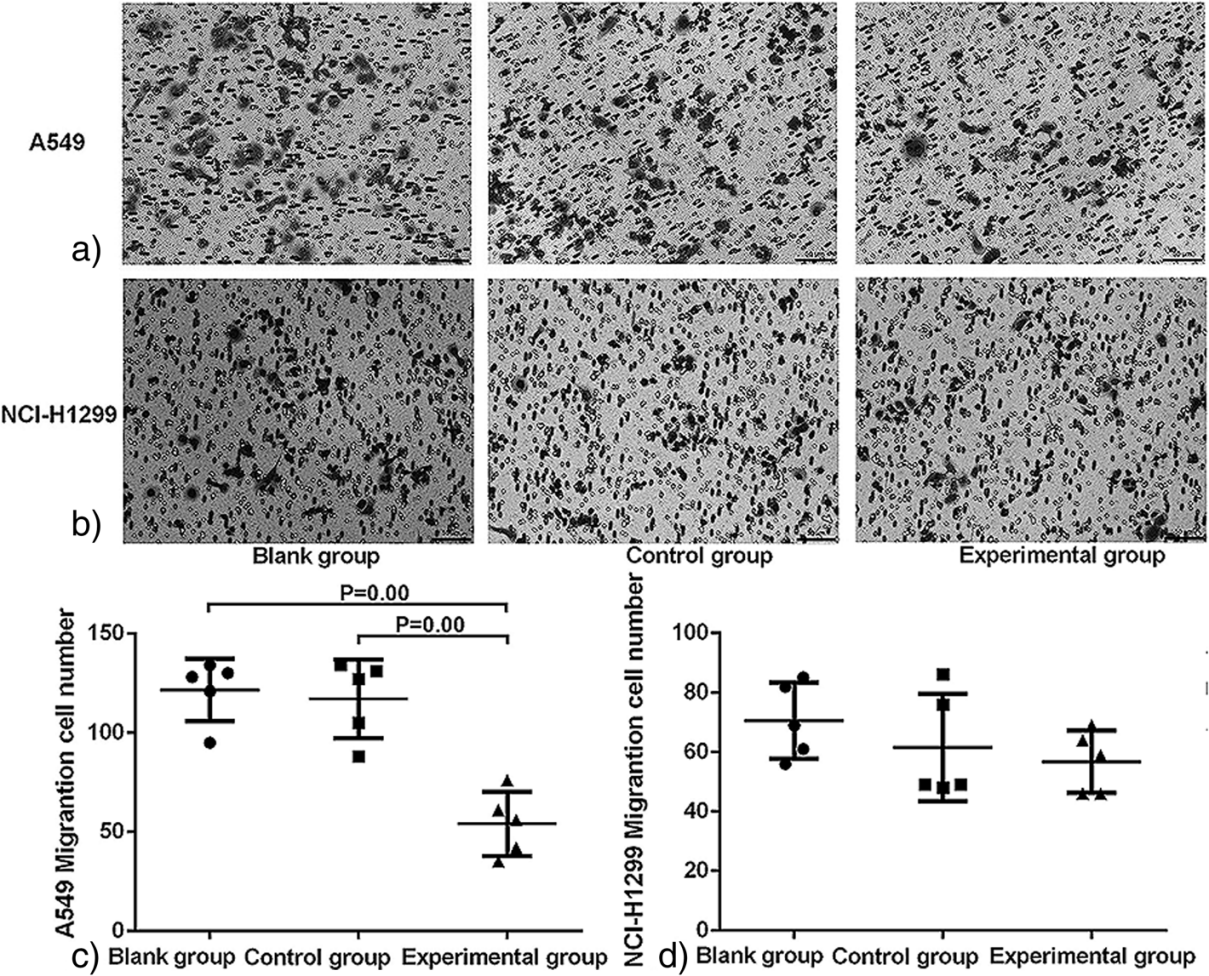
Inhibition of FASN suppresses the migration and invasion ability of A549 cells. A549 (**a**) and NCI-H1299 (**b**) cells were seeded into the upper chamber of a transwell system. The cells in the lower chamber were stained and counted to assess the migration and invasion ability of the target cells. Compared with the blank and control groups, the numbers of A549 cells were significantly lower (**c**); however, there were no statistically significant differences between the numbers of NCI-1299 cells in the experimental group and the other groups (**d**)

To check whether FASN deficiency affects the malignant biological behavior of NSCLC cells, the proliferation, migration, and invasion of NSCLC cells were detected by CCK8 and transwell assay after FASN inhibition. The CCK8 results show that the viability of A549 and NCI-H1299 cells on the fifth day after transfection with FASN-siRNA was significantly lower than in the corresponding blank and control group cells (*P* < 0.05; Fig. 4a, b). As exhibited by the transwell assay, the number of A549 experimental group cells was 54 ± 16.14, which was significantly lower than for the A549 blank group and the A549 control group (*P* < 0.00; Fig. 5a, b). Interestingly, there was no statistically significant difference in the numbers of migrating cells in the NCI-H1299 experimental group and corresponding blank or control groups (Fig. 5c, d).

### Inhibition of FASN blocks xenograft tumor growth of NSCLC in nu/nu mice

**Fig. 6:**
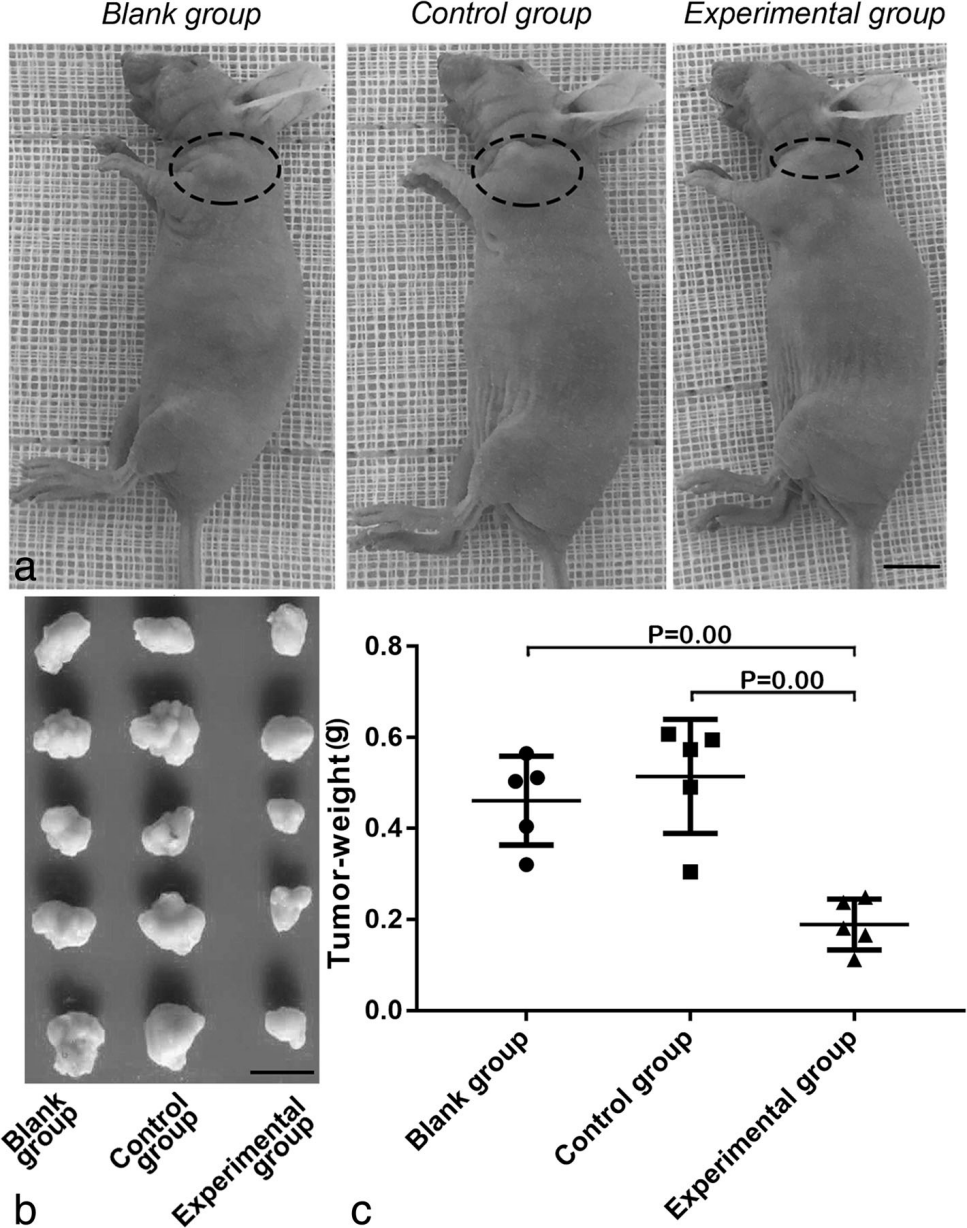
Inhibition of FASN blocks xenograft tumor growth of NSCLC in nu/nu mice. A preliminary experiment was conducted to detect the tumorigenicity ability of wild-type A549 cells (**a**). Three groups of A549 cells were subcutaneously injected into nu/nu mice and xenograft tumors were harvested after 3 weeks (**b**), The tumor weight of the experimental group was significantly lower than those of the blank and control groups (**c**). The scale bars of (**a**) and (**b**) is 1 cm

The xenograft tumor model was used to determine the effect of FASN inhibition in NSCLC progress. Investigation of the tumorigenicity of A549 and NCI-H1299 cells indicated that NCI-H1299 cells had poorer tumorigenicity than A549 cells. As a result, A549 cells were selected for the xenograft tumor model (Fig. 6a). The three groups of A549 cells were collected and injected into nu/nu mice, with tumors isolated and weighed after 3 weeks. Tumor weights for the control and blank groups were 0.51 ± 0.13 g and 0.46 ± 0.10 g, respectively. However, the tumor weight for the A549 experimental group was 0.19 ± 0.06 g, significantly lower than for the control and blank groups (*P* = 0.00 and *P* = 0.00, respectively; Fig. 6b, c). There was no significant difference in tumor weight between the control and blank groups. These findings indicate that inhibition of FASN blocks xenograft tumor growth of NSCLC in vivo under experimental conditions.

## Discussion

In 1994, FASN (known as antigen OA-519) was identified as a prognostic molecule for breast cancer patients with obviously poor prognoses [30]. FASN is a rate-limiting enzyme of de novo fatty acid synthesis and the lipid synthetic pathway; normal cells have lower FASN expression due to the inactivation of endogenous fatty acid metabolism. Recently, overactivated FASN has been found in different types of cancers [31]. FASN is a multifunctional enzyme, and its main physiological function is to supply lipids for membrane production and the synthesis of fatty acids. Previous studies have suggested that FASN expression and its activity contribute to the proliferation, metastasis, migration, and invasion of cancer cells [32]. The inhibition of FASN significantly improves the malignant biological behavior of many cancer cell lines [33].

In this study, inhibition of FASN by FASN RNAi significantly suppressed the proliferation of two types of NSCLC cells: A549 and NCI-H1299. However, inhibition of FASN suppressed the migration and invasion of A549 cells, but not NCI-H1299 cells. These results suggest that FASN could be a key regulator of migration and invasion in NSCLC cells, but it is not ubiquitous in all NSCLC cells since A549 was isolated from primary lung cancer while NCI-H1299 was isolated from metastatic lung cancer (lymph node) and contained the NRAS gene mutant. To better understand the effects of FASN in the tumor growth of NSCLC cells, a xenograft tumor model was constructed. The results show that inhibition of FASN blocked xenograft tumor growth of NSCLC in vivo, suggesting that FASN contributes to the malignant biological behavior of NSCLC.

Aerobic glycolysis and de novo lipid biosynthesis are overactive in most malignant tumors. These overactive metabolic factors provide adequate energy and material intermediates for the proliferation, metastasis, migration, and invasion of cancer cells [34–36]. Glucose metabolism provides acetyl-CoA for de novo fatty acid synthesis, which could be regulated by FASN. However, it remains unclear whether FASN functions as a regulator in the glucose metabolism of NSCLC. AZGP1 plays a key role in lipid mobilization [37] and its expression may be regarded as a prognostic biomarker of NSCLC [38]. AZGP1 mRNA expression in human lung tissue has been found to correlate with the stage of lung cancer disease [39]. Additionally, activation of Akt/ERK pathways has been found in most NSCLC tissues and is related to tumor growth, apoptosis, and drug resistance in lung cancer [40–42]. However, the relationship between the FASN and Akt/ERK pathways remains poorly understood in NSCLC. As a result, this study also determined the expression of target molecules and the level of glucose metabolism activity.

The present study determined Akt/ERK pathway and glucose metabolic activities under FASN deficiency. RT-qPCR and western-blot results show that inhibition of FASN suppressed the expression of phosph-Akt and phosph-ERK for the two types of NSCLC cells. Simultaneously, the expressions of PKM2, which are key enzymes of glucose metabolism, were also decreased in the two types of NSCLC cells. Additionally, the levels of ATP and lactic acid were detected to evaluate glucose metabolism after loss of FASN expression, with the results showing that inhibition of FASN suppressed ATP and lactic acid levels. These findings imply that FASN can be a key regulator in glucose metabolism via the Akt/ERK pathways. However, a clearer understanding may be obtained by conducting studies that recover Akt or PKM2 inhibitors.

## Conclusions

The present study demonstrates that FASN deficiency clearly inhibits the malignant phenotypes of lung cancer cells in vivo and in vitro. Additionally, suppression of FASN reduces the activities of glucose metabolism and the AKT/ERK pathway. Overall, inhibition of FASN suppresses malignant biological behavior, glucose metabolism but also the activity of the Akt/ERK pathway of NSCLC cells. FASN could be a key factor that involves in the AKT/ERK pathway activity, glucose metabolism and even altered the malignant phenotype in lung cancer cells. These findings yield profound insights into the functions and relationships of FASN, the Akt/ERK pathway, and glucose metabolism in NSCLC.

## Abbreviations

Akt: Protein Kinase B
AZGP1: Alpha2-Zine-Glycoprotein1
ERK: Extracellular signal regulated kinase
HK2: Human kallikrein 2
mTOR: mammalian target of rapamycin
PKM2: Embryonic pyruvate kinase M2
